# Comparison of the Fecal Microbiota from Long-term Captive and Newly Captured Whale Sharks (*Rhincodon typus*)

**DOI:** 10.1264/jsme2.ME25023

**Published:** 2025-09-30

**Authors:** Takaomi Ito, Takao Segawa, Kazuto Takasaki, Takahiro Matsudaira, Itsuki Kiyatake, Hiroyuki Irino, Yu Nakajima

**Affiliations:** 1 Osaka Aquarium KAIYUKAN, 1–1–10 Kaigandori, Minato-ku, Osaka 5520022, Japan; 2 Nihon University Veterinary Research Center, 1866 Kameino, Fujisawa, Kanagawa, 2520880, Japan; 3 FASMAC CO., LTD., 5–1–3 Midorigaoka, Atsugi, Kanagawa, 2430041, Japan; 4 Japan Agency for Marine-Earth Science and Technology (JAMSTEC), Research Institute for Marine Resource Utilization (MRU), 2–15 Natsushima-cho, Yokosuka, Kanagawa, 2370061, Japan

**Keywords:** microbiota of elasmobranch, long-term captive effect, shark health assessment, genus *Ureaplasma*

## Abstract

Despite its ecological importance, the gut microbiota of whale sharks (*Rhincodon typus*) remains poorly understood. Therefore, the present study exami­ned how environmental differences affect the fecal microbiota by comparing long-term captive and newly captured individuals. Fecal samples were collected over time from four long-term captive and two newly captured whale sharks, with seawater also being sampled from their respective tanks. Using 16S rRNA sequencing, 12,497 amplicon sequence variants (ASVs) were identified, including 6,976 classified as major ASVs. There were no significant differences in alpha diversity indexes between long-term captive and newly captured sharks; however, the latter showed slightly larger variance in four indexes. The ASV count per individual was slightly lower in long-term captive sharks than in their newly captured counterparts. In long-term captive individuals, *Photobacterium* was highly abundant. Conversely, *Ureaplasma* was dominant in newly captured individuals, but was barely detected in long-term captive sharks. Although alpha diversity did not differ significantly between the groups, a beta diversity ana­lysis showed clear distinctions. The high abundance of *Ureaplasma* in newly captured sharks suggests its involvement in nitrogen metabolism, possibly through urea recycling. Although further research is needed to clarify the taxonomic position and ecological functions of these *Ureaplasma* populations, the present study provides key insights for the conservation of wild whale sharks and improving health management for captive individuals.

In recent years, research on symbiotic microorganisms using amplicon sequencing and metagenomic ana­lyses has rapidly advanced. Microbial communities play vital roles in supplementing host metabolism, preventing pathogenic infections, and enhancing immune function, making them essential for maintaining host health (Young, 2017). Disruptions in the microbial balance are also linked to the onset of non-infectious diseases (Lloyd-Price *et al.*, 2016; Gilbert *et al.*, 2018). Gut microbiota research is particularly active because the colon hosts more than 100 trillion microbes that form complex interrelationships, which contribute to metabolism, nutrition, and immunity (Guinane and Cotter, 2013; Kho and Lal, 2018).

In fish microbiota studies, the skin, gills, and intestines are key areas of interest. The skin and gills, in direct contact with the environment, are protected by scales, mucus, and immune substances (Esteban, 2012; Dash *et al.*, 2018; Reverter *et al.*, 2018). Additionally, microbiota in these areas may help prevent opportunistic infections, regulate immune responses, and promote wound healing (Pratte *et al.*, 2018; Legrand *et al.*, 2020; Wang *et al.*, 2023). However, microbiota have been studied more extensively in the gut than in the skin and gills. In commercial fish species, metagenomic research methods are used to investigate the gut microbiota’s effects on host physiology, factors shaping its composition, its immunological effects, and antimicrobial resistance mechanisms (Tyagi *et al.*, 2019; Yukgehnaish *et al.*, 2020; Nallaiah *et al.*, 2022).

Elasmobranchs, which comprise the class Chondrichthyes in fish, have limited commercial value; therefore, very few studies have been performed on their microbiota. As of 2021, the microbiota in 41 elasmobranch species across various body sites, including the oral cavity, skin, gills, blood, and intestines, has been reported (Costa *et al.*, 2022). Perry *et al.* (2021) characterized the gut microbiota of elasmobranchs as having the following: 1) lower alpha diversity than the gut microbiota of other vertebrates, 2) a core microbiota dominated by *Photobacterium*, 3) common genera, such as *Cetobacterium*, *Clostridium*, and *Campylobacter*, 4) some hosts with a high abundance of unknown taxa, 5) unclear details on the effects of the gut location, internal anatomy, and feeding behavior/diet, and 6) the potential for some host species to perform plant degradation/fermentation. These findings suggest that data from other vertebrates, including humans, cannot be directly extrapolated to elasmobranchs. Elasmobranch populations have been declining globally due to overfishing, increased bycatch rates, and their low resilience to fishing pressures (Lucifora *et al.*, 2011; Dulvy *et al.*, 2017). Given their wide distribution and ecological and conservation importance as apex predators in the marine food chain, there is an urgent need to accumulate knowledge on the gut microbiota of elasmobranchs, which may significantly impact their health.

The gut microbiota of teleosts is known to vary depending on the host species (Riiser *et al.*, 2020), individual (Nielsen *et al.*, 2017), habitat (Kim *et al.*, 2021), life stage (Verner-Jeffreys *et al.*, 2003; Romero and Navarrete, 2006), diet (Deck *et al.*, 2023; Józefiak *et al.*, 2023), fasting (Parris *et al.*, 2019; Wang *et al.*, 2024), and various other environmental factors (Talwar *et al.*, 2018; Parata *et al.*, 2020; Yukgehnaish *et al.*, 2020). Changes in the rearing environment of Atlantic salmon (*Salmo salar*) have significantly affected the alpha and beta diversities of the gut microbiota, indicating that the gut microbiota possesses plasticity and resilience (Uren Webster *et al.*, 2020). Additionally, in many teleosts, such as cavefish (*Sinocyclocheilus* species), the common carp (*Cyprinus carpio*), Trinidadian guppy (*Poecilia reticulata*), and tropical gar (*Atractosteus tropicus*), the gut microbiota significantly changes when they are reared in artificial environments from that in the wild (Dhanasiri *et al.*, 2011; Sullam *et al.*, 2015; Eichmiller *et al.*, 2016; Tan *et al.*, 2019; Méndez-Pérez *et al.*, 2020; Zhou *et al.*, 2022). On the other hand, limited information is currently available on differences in the microbiota, including that of the gut, between wild and captive elasmobranchs.

Whale sharks (*Rhincodon typus*), the sole species of the genus *Rhincodon* and the world’s largest fish, grow up to 14‍ ‍m in length (Chen *et al.*, 1997). They inhabit tropical to temperate oceanic regions (Colman, 1997) and primarily filter-feed on copepods, crustaceans, and fish larvae using gill rakers in their mouths (Kamal *et al.*, 2016; Manuhutu *et al.*, 2020). With an estimated lifespan of 70–80 years and an ovoviviparous reproductive mode requiring long gestation, whale sharks struggle to adapt quickly to habitat destruction caused by human activity and climate change; therefore, they face challenges in recovering from population declines (Norman, 2004). The IUCN Red List classifies whale sharks as endangered, with a declining population (IUCN, 2024), and the Convention on International Trade in Endangered Species of Wild Fauna and Flora lists them under Appendix II (CITES, 2024). Therefore, there is an urgent need for fundamental data to support whale shark conservation. However, whale shark microbiota research is limited to studies on the microbiota from various body parts of specific individuals and the gut microbiota in fecal samples from five wild sharks (Doane *et al.*, 2020; Pratte *et al.*, 2022).

At Osaka Aquarium KAIYUKAN, wild whale sharks are kept in captivity for extended periods, allowing research on their physiology, behavior, and ecology. After a certain period in captivity, all whale sharks are released into the wild with pop-up satellite archival tags or video loggers to study their behavior. In the present study, we hypothesized that newly captured whale sharks retain the fecal microbiota of their counterparts in the wild. The primary objective was to gain insights into how environmental differences affect the fecal microbiota by comparing newly captured and long-term captive individuals. Specifically, the following inves­tigations were conducted: 1) assessing the taxonomic composition of amplicon sequence variants (ASVs) in fecal and seawater microbiota, 2) comparing the fecal and seawater microbiota in long-term captive whale sharks, and 3) comparing the fecal microbiota between long-term captive and newly captured whale sharks.

## Materials and Methods

### Sample collection

**Fig. 1.**
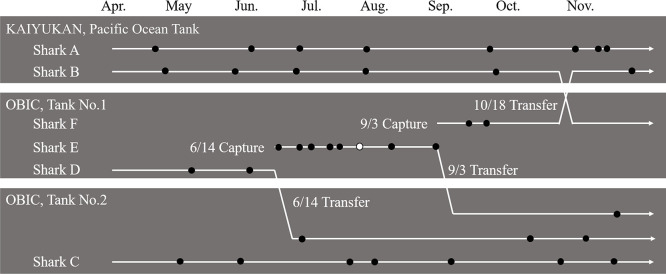
Fecal sampling schedule for long-term captive and newly captured whale sharks (*Rhincodon typus*). Black circles indicate dates when fecal and seawater samples were both collected. In contrast, the white circle represents the sampling of feces only from Shark E on July 24.

Between April and November 2019, six whale sharks housed at Osaka Aquarium KAIYUKAN and its affiliated facility, the Osaka Aquarium Biological Institute of Iburi Center (OBIC), were exami­ned. All six sharks were captured in set nets off the coast of Kochi Prefecture. To ensure proper management and compatibility, whale sharks were occasionally moved between tanks. Their diet primarily consisted of krill and mysid shrimp, supplemented with Sakura shrimp, whitebait, and artificial shark feed, provided twice daily at fixed times. Feeding amounts were adjusted based on each shark’s length and condition, averaging 3.4–7.0‍ ‍kg day^–1^ per shark. On some days, feeding was skipped for operational reasons. Throughout the captive period at both facilities, the sharks received no medical treatments or medications. Table 1 details the sampled sharks, and Fig. 1 shows sampling periods and tank movements.

At Osaka Aquarium KAIYUKAN, sharks were housed in the Pacific tank (5,400 m^3^ and 9-m deep). At OBIC, they were kept in either the first tank (1,000 m^3^ and 5-m deep) or the second tank (3,000 m^3^ and 5-m deep). The Pacific tank contains seawater transported by a seawater transport vessel from 100‍ ‍km south of Osaka Aquarium KAIYUKAN, off the coast of Hinomisaki, Wakayama Prefecture. OBIC tanks contain well water treated for manganese removal. Both facilities operate semi-closed recirculating systems with filtration and ozone treatment for water clarity and sterilization. Water temperature is maintained year-round at 23°C in the Pacific tank, and averages 24.4°C (22.5–26.0°C) and 24.8°C (23.1–26.7°C) in the first and second OBIC tanks, respectively. Daily water quality tests measured salinity, pH, dissolved oxygen, ammonia, and nitrite, with no abnormal values being detected during the study.

Fecal samples were collected from four long-term captive whale sharks (Sharks A to D) and two newly captured whale sharks (Sharks E and F) caught near OBIC and brought into captivity. Fecal samples were collected through direct observations. When defecation was observed, staff retrieved the feces immediately. Fecal samples were taken from internal fecal matter that had not contacted seawater. Additionally, seawater from the tank was collected separately from feces, except on July 24, 2019, when only fecal samples from Shark E were collected. Water samples were collected using the previously described filtration method (Minamoto *et al.*, 2021). After rinsing plastic bottles several times‍ ‍using seawater in the aquarium, they were filled with 2 L of seawater. Poured seawater was filtered with Sterivex-GP (Merck Millipore) through an electric pump. The filters were kept frozen until extraction. Fecal and seawater samples were both immediately frozen at –30°C after collection. During the study, 3–9 sets of fecal and seawater samples were collected from each individual. All animal procedures were approved by the KAIYUKAN and NIFREL Research Ethics Review Committee (permit number: KN24012).

### DNA extraction and 16S rRNA sequencing

Total DNA was extracted from 73 samples: 37 fecal samples using the ISOSPIN Fecal DNA Kit (Nippongene) and 36 seawater samples using the DNeasy Blood & Tissue Kit (QIAGEN), according to the manufacturers’ protocols (Minamoto *et al.*, 2021). The 16S rRNA gene region used for a bacterial community anal­ysis was amplified with the Bact-0341 (5′-CCTACGGGNGGCWGCAG-3′) and Bact-0785 (5′-GACTACHVGGGTATCTAATCC-3′) primers (Klindworth *et al.*, 2013). Polymerase chain reaction amplification was performed using 2× KAPA HiFi Hot Start ReadyMix (KAPA Biosystems) according to the manufacturer’s instructions and the Illumina library preparation protocol (https://support.illumina.com/downloads/16s_metagenomic_sequencing_library_preparation.html). Libraries were quantified using a Qubit fluorometer with the Qubit dsDNA HS Assay Kit (Thermo Fisher Scientific), and the library size was assessed using an Agilent BioAnalyzer with high-sensitivity DNA chips (Agilent Technologies). Equimolar concentrations of libraries were pooled for sequencing.

In total, 6,688,275 reads (average: 91,620; max: 143,679; minimum: 62,804; standard deviation: 16,363) of 16S rRNA amplicons were generated using an Illumina MiSeq sequencer (Illumina) with the V3 region, producing 2×300-bp paired-end reads. Initial sequence processing was performed using MiSeq Reporter software version 1.3.17.0 (Illumina) to demultiplex samples and remove adapters and primer sequences. Data were then exported in the FASTQ format. Sequencing was performed by FASMAC, with sequencing data available in the NCBI Sequence Read Archive under the BioProject ID PRJNA1226372.

### Data and statistical ana­lyses

Raw reads underwent quality control via fastp v.0.23.4 (Chen *et al.*, 2018) using the parameters “-q 30 -n 1 -t 5 -T 5 -l 100”. After trimming, sequences were processed in QIIME2 version 2024.5.0 (Bolyen *et al.*, 2019). DADA2 denoising was applied with the parameters “--p-trim-left-f 3 --p-trim-left-r 3 --p-trunc-len-f 275 --‍p-trunc-len-r 250 --p-n-reads-learn 4,000 --p-min-overlap 10.” ASVs were taxonomically assigned at the genus level or higher using the Genome Taxonomy Database (GTDB) (Parks *et al.*, 2022) Release 220 to assess the 16S rRNA gene. Only taxa (genera) with a read frequency ≥0.1% in at least one individual were included (major ASVs).

Statistical ana­lyses were conducted using QIIME2’s q2-diversity plugin, which computes alpha and beta diversities metrics, performs related statistical tests, and generates visualizations. Alpha diversity was assessed via the observed features, the Shannon index, Pielou evenness, and Faith PD index values. The Kruskal–Wallis test in QIIME2 was used to compare alpha diversity indices. Beta diversity was analyzed via a principal coordinate ana­lysis (PCoA) using Jaccard, Bray–Curtis, unweighted unique fraction (UniFrac), and weighted UniFrac phylogenetic distances, with significance being tested through a permutational multivariate anal­ysis of variance (PERMANOVA) in QIIME2. All alpha and beta diversities data were obtained from outputs generated via “QIIME2 view” online software (https://view.qiime2.org/).

## Results

### Taxonomic composition of ASVs in fecal and seawater microbiota

**Fig. 2.**
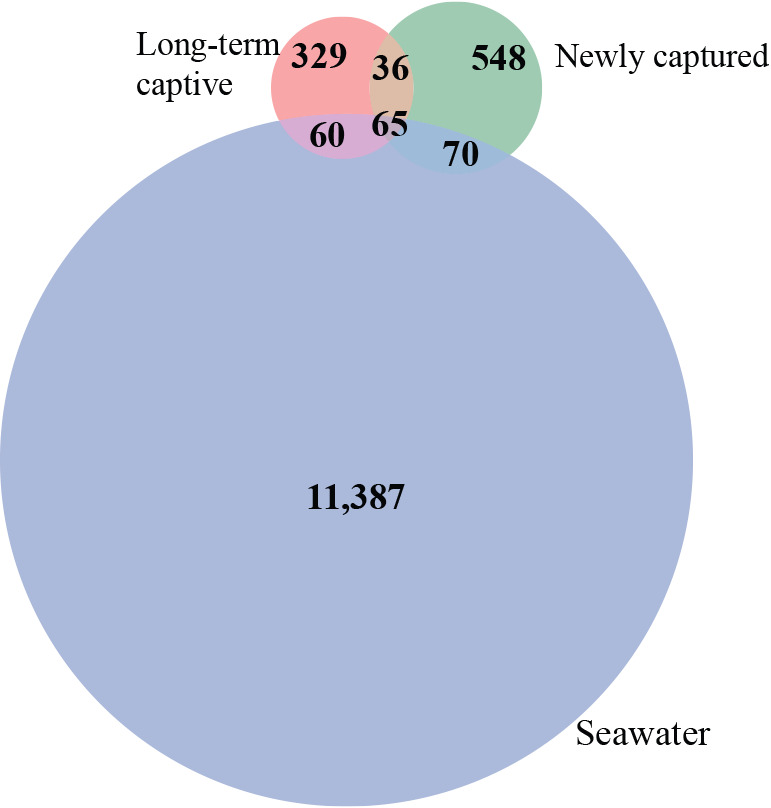
Number of shared ASVs among whale shark fecal samples and seawater samples from holding tanks. A Venn diagram showing the sharing of all ASVs found in 37 fecal and 36 seawater samples. Fecal samples were distinguished by captive length.

In the 16S rRNA amplicon sequencing of 37 fecal samples from long-term captive and newly captured whale sharks, along with 36 seawater samples from holding tanks, 1,178,987 reads were assigned to 12,497 ASVs. Of these, 6,976 ASVs reached more than 0.1% relative abundance (Table S1). The number of ASVs per individual was 56.0 (range: 29–121; standard deviation: 18.0) in long-term captive sharks and 61.6 (range: 17–201; standard deviation: 74.0) in newly captured individuals (Table S2). In total, 101 ASVs were shared between the two whale shark groups, while 65 were shared between fecal samples and seawater from the holding tanks (Fig. 2).

At the phylum level, 11 phyla were identified in fecal samples from long-term captive whale sharks, primarily Bacillota_A (58.7% [range: 14.9%–98.6%]), *Pseudomonadota* (32.9% [0.86–76.0%]), and *Fusobacteriota* (5.20% [0%–47.7%]). In newly captured whale sharks, 28 phyla were detected, with Bacillota_I (62.1% [1.05%–99.8%]) and *Pseudomonadota* (7.30% [0%–47.9%]) being the most abundant (Fig. S1 and Table S3). Although ASVs assigned to unclassified Bacteria (GTDB classification: d__Bacteria;__) accounted for an average of 20.9% (max: 96.7; min: 0, standard deviation: 35.1) in newly captured sharks, ASVs showing very large relative abundance in a few samples were of eukaryotic mitochondrial origin with 80–90% identity to *Eimeria nieschulzi* and *Cyclospora cayetanensis*. Other ASVs with less than 0.1% per sample were unclassified Bacteria. The seawater microbiota from holding tanks mainly consisted of *Pseudomonadota* (59.3% [20.9%–90.5%]), *Bacteroidota* (18.7% [3.36%–59.6%]), and *Patescibacteria* (3.2% [0.30%–16.5%]), with 56 phyla being detected.

**Fig. 3.**
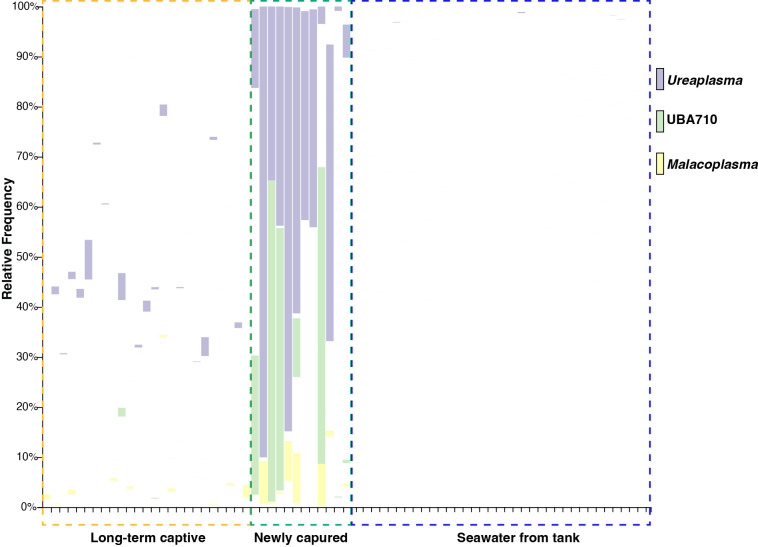
Relative abundance of three key genera in fecal and seawater microbiota. Each bar represents each sample. Purple, green, and yellow bars indicate the genera *Ureaplasma*, UBA710, and *Malacoplasma* according to the GTDB R220 classification, respectively.

At the genus level, the dominant genera in fecal samples from long-term captive whale sharks were *Photobacterium* (average: 31.1%), UBA866 (23.5%), Romboutsia_A (15.2%), and *Tyzzerella* (5.94%), whereas these taxa were present at very low levels in newly captured individuals (1.10, <0.10, 2.74, and 1.50%, respectively; Table S4). Instead, newly captured whale sharks exhibited a high relative abundance of *Ureaplasma* (average: 40.4%), UBA710 (18.0%), and *Malacoplasma* (3.12%). These three genera, classified within the phylum Bacillota_I (class Bacilli_A; order *Mycoplasmatales*), were detected at low levels (<0.1–1.0%) in the feces of long-term captive individuals (Fig. 3).

### Comparison of fecal and seawater microbiota in long-term captive whale sharks

**Fig. 4.**
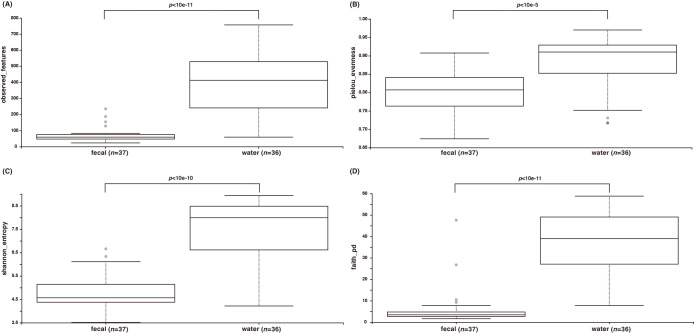
Differences in alpha diversities between fecal samples and seawater samples. Observed ASVs (A), Pielou’s evenness (B), the Shannon index (C), and Faith’s PD index (D).

**Fig. 5.**
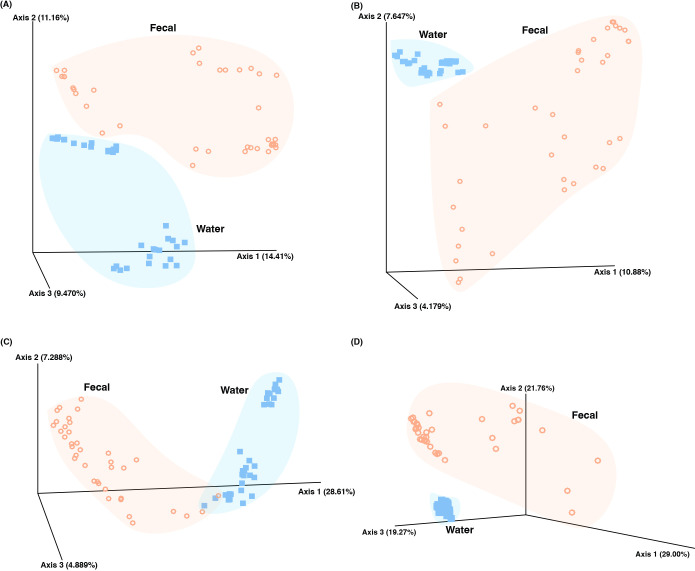
Principal coordinates ana­lysis of fecal and seawater samples. Beta Diversity metrics. The Bray–Curtis index (A), Jaccard dissimilarity index (B), unweighted UniFrac (C), and weighted UniFrac (D). Individual points represent each sample (orange: fecal samples; blue: seawater samples). Axes represent the percentage of variation explained.

An alpha diversity ana­lysis revealed that the seawater microbiota exhibited significantly higher diversity than the fecal microbiota across all indices, including the Shannon index, Faith’s PD, and Pielou’s evenness (*P*<0.001; Fig. 4). To assess the relationship between fecal and seawater microbiota, beta diversity ana­lyses were conducted using PCoA and PERMANOVA (Fig. 5). PCoA revealed distinct clustering between fecal and seawater microbiota across all metrics, including the Jaccard index, Bray–Curtis index, and unweighted and weighted UniFrac. PERMANOVA confirmed significant differences (*P*<0.01) between the two groups across all metrics.

### Comparison of the fecal microbiota between long-term captive and newly captured whale sharks

**Fig. 6.**
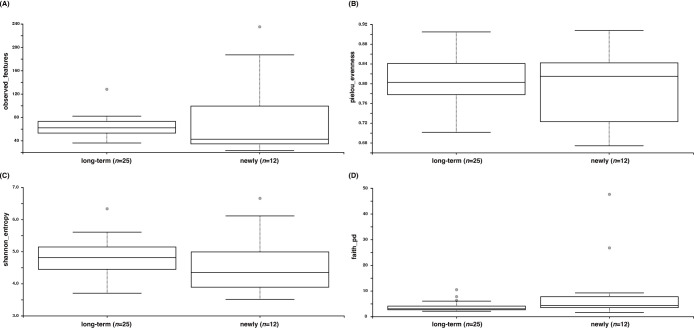
Differences in alpha diversities between fecal samples from long-term captive and newly captured whale sharks. Observed ASVs (A), Pielou’s evenness (B), the Shannon index (C), and Faith’s PD index (D).

**Fig. 7.**
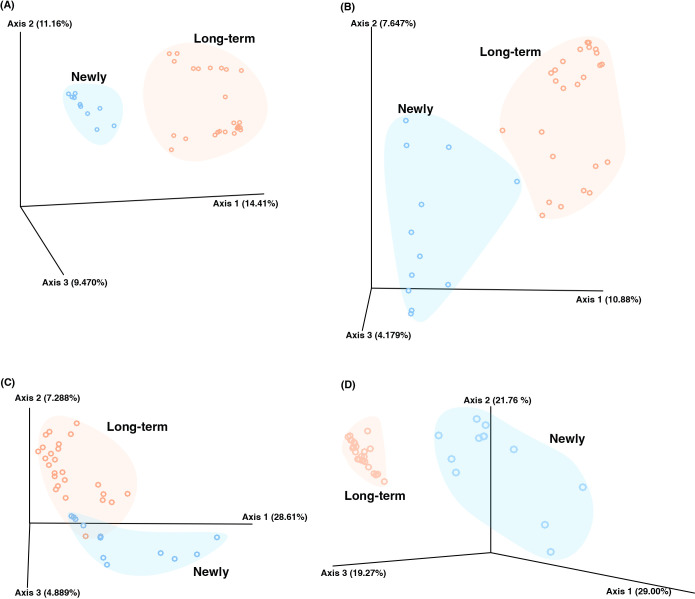
Principal coordinates ana­lysis of fecal samples from long-term captive and newly captured whale sharks. Beta diversity metrics: the Bray–Curtis index (A), Jaccard dissimilarity index (B), unweighted UniFrac (C), and weighted UniFrac (D). Individual points represent each sample (orange: long-term captive; blue: newly captured). Axes represent the percentage of variation explained.

Alpha diversity indices, including observed features, Faith’s PD, the Shannon index, and Pielou’s evenness, showed no significant differences (*P*>0.05) between the fecal microbiota of long-term captive and newly captured whale sharks (Fig. 6). However, a beta diversity ana­lysis showed distinct clustering between the two groups in PCoA across all metrics, namely, the Jaccard index, Bray–Curtis index, and unweighted and weighted UniFrac (Fig. 7). A PERMANOVA ana­lysis confirmed that the composition of the fecal microbiota significantly differed (*P*<0.01) between long-term captive and newly captured whale sharks across all four beta diversity indices.

When tracking temporal changes in the microbiota for each individual, *Pseudomonadota* and *Bacillota* were stable as dominant groups in long-term captive sharks (Sharks A to D). On the other hand, newly captured sharks (Sharks E and F), particularly Shark E, showed apparent major changes 1 and 3 months after their capture. However, marked changes over several months were not observed in Shark F (Fig. S2).

## Discussion

Fecal sample collection from aquatic animals in the wild is extremely challenging (Hunt *et al.*, 2019; May and El‐Sabaawi, 2022). This difficulty primarily arises from the unpredictability of defecation behavior and the rapid dispersion of feces in the water column. Even in captivity, fecal sampling remains difficult for large-bodied or husbandry-sensitive species, which are often challenging to maintain under stable conditions. In shared tanks, feces may be disturbed by other animals or quickly removed by recirculating filtration systems, further hindering sample collection. At our facility, we leveraged our extensive experience in whale shark husbandry to accurately detect the timing of defecation and thereby attempted to collect fresh fecal samples. In the present study, the minimal number of shared ASVs, along with the results of statistical ana­lyses of diversity indices, suggested that contamination from captive seawater during sample collection had a negligible effect on the study results. In addition, the generally solid consistency of whale shark feces is likely to have further reduced the risk of contamination.

Our facility also houses other planktivorous elasmobranchs, such as the reef manta ray (*Mobula alfredi*) and the spinetail devil ray (*Mobula mobular*), the feces of which typically have a watery consistency. To the best of our knowledge, due to the difficulty associated with collecting watery feces, no studies on the gut microbiota of these planktivorous rays have been published. The majority of studies on planktivorous fishes have focused on teleosts. The gut microbiota of marine planktivorous teleosts was previously shown to be dominated by gammaproteobacteria and betaproteobacteria lineages, such as the genera *Vibrio*, *Pseudomonas*, *Psychrobacter*, *Achromobacter*, *Shewanella*, *Alteromonas*, and *Endozoicomonas* (Egerton *et al.*, 2018; Kavazos *et al.*, 2022). In comparisons with these teleosts, the fecal microbiota observed in whale sharks in the present‍ ‍study showed a clearly distinct composition. These differences may reflect not only host-related factors, such as‍ ‍physiology, gastrointestinal conditions, and diet, but also‍ ‍external factors, including environmental microbial exposure, the captivity status, and methodological variations. Future comparative ana­lyses involving other planktivorous elasmobranchs or teleosts fed similar diets will be valuable for establishing whether the composition of the microbiota observed in this study is specific to whale sharks.

To further examine possible sources of variation in the whale shark microbiota, a relevant comparison may be conducted with the study by Pratte *et al.* (2022), which investigated the microbiota in both wild and captive individuals. They analyzed bacterial communities from the gills, skin, cloaca, and feces of wild individuals from three geographically distinct locations (Maldives, Tanzania, and St. Helena) as well as from one captive whale shark. Although sufficient samples were collected from the gills, skin, and cloaca, only five fecal samples from wild individuals were subjected to 16S rRNA amplicon sequencing. They found <90 ASVs per sample, with dominant genera including *Photobacterium* and *Tyzzerella*, along with taxa from *Clostridia*, *Campylobacter*, and *Fusobacteria*. The present study identified a similar number of ASVs in newly captured whale sharks. However, despite the geographic separation of the locations sampled by Pratte *et al.* (2022), they reported minimal variations in the dominant bacterial taxa across sites. Conversely, although the genera *Photobacterium* and *Tyzzerella* were abundant in a few of our samples, we observed a lower relative abundance of *Clostridia*, *Campylobacter*, and *Fusobacteria* than their study.

One key difference between the whale sharks sampled by Pratte *et al.* (2022) and those in the present study may be their behavior. The regions sampled in their study are known feeding aggregation hotspots for whale sharks (Riley *et al.*, 2010; Rohner *et al.*, 2015, 2020; Perry *et al.*, 2020). For example, in the Gulf of Mexico, whale sharks continuously ram-filter near the surface (Hoffmayer *et al.*, 2007). In contrast, whale sharks migrating through Japanese coastal waters travel northward along the Kuroshio Current from tropical regions in search of prey, temporarily residing in nutrient-rich warm waters in the Kuroshio–Oyashio transition zone (~1,000‍ ‍km northeast of Kochi Prefecture). As sea temperatures fall in autumn, they leave Japanese waters (Matsunaga *et al.*, 2003). Fish are known to reduce feeding activity during migration (Larsen, 1980; Navarro and Gutiérrez, 1995; Fan *et al.*, 2019; Hvas, 2022). Moreover, whale sharks migrating from tropical waters to Japan may undergo periods of prolonged starvation, as suggested by the findings of multiple stable isotope and blood biochemical ana­lyses (Wyatt *et al.*, 2019; Ito and Segawa, 2025), with starvation markedly affecting the composition of the gut microbiota (Xia *et al.*, 2014; Liu *et al.*, 2020; Zou *et al.*, 2023). In addition, stress associated with prolonged starvation and environmental changes may cause dysbiosis, which needs to be considered when interpreting microbiota profiles. Therefore, we infer that the fecal microbiota of whale sharks inhabiting feeding aggregations markedly differs from that of the migrating individuals sampled in our study due to differences in feeding conditions. Specifically, the microbiota identified by Pratte *et al.* (2022) may reflect a diet rich in resources, whereas the microbiota observed in the present study may reflect the effects of long-term starvation and stress in the wild. In support of this notion, we found that the fecal microbiota of long-term captive whale sharks, which were fed regularly, exhibited a high relative abundance of genera similar to those detected by Pratte *et al.* (2022), *e.g.*, *Photobacterium* and *Tyzzerella*.

The composition and diversity of the fecal microbiota of newly captured and long-term captive whale sharks significantly differed. Although numerous studies have compared the gut microbiota between wild and captive teleosts (Méndez-Pérez *et al.*, 2020; Uren Webster *et al.*, 2020; Zhou *et al.*, 2022), research on elasmobranch species remains limited. One study on bonnethead sharks (*Sphyrna tiburo*) found no significant differences in alpha or beta diversities between wild individuals and those kept in short-term captivity (3 weeks) (Leigh *et al.*, 2021). Clavere-Graciette *et al.* (2022) exami­ned the cloacal microbiota of whitespotted eagle rays (*Aetobatus narinari*), comparing wild individuals with long-term captive individuals fed a similar diet to that of wild conspecifics. Although they found significant differences in alpha and beta diversities, overall differences were minimal. In these two cases, the lack of pronounced differences in the microbiota may be attributed to the short captivity duration in the former study and similarities in the diet to wild individuals in the latter study. In contrast, the present results suggest that the fecal microbiota of newly captured whale sharks reflects a starvation state at the time of capture. However, after transitioning to captivity and receiving feed regularly, based on the results obtained from long-term and newly captured whale sharks, the gut microbiota was considered to have undergone marked changes. Nevertheless, temporal changes in the microbiota for Sharks E and F did not sufficiently support this conclusion. These dietary adjustments may have contributed to the marked differences observed between the fecal microbiota of newly captured and long-term captive whale sharks, and this hypothesis may be clarified through future longer-term individual-focused ana­lyses.

A notable result in this study was the high abundance of the genus *Ureaplasma* in the fecal microbiota of newly captured whale sharks. *Ureaplasma*, a member of the phylum *Mycoplasmatota*, is the smallest self-replicating bacterium, characterized by its lack of a cell wall. Although uncultured bacterial genomes from fish have been classified as being closely related to *Ureaplasma* in the GTDB database, its isolation from poikilotherms has yet to be confirmed. Considering the potential role of this unknown *Ureaplasma* in whale sharks, a key observation was its markedly low abundance in fecal samples from long-term captive individuals (Fig. 3). In a metagenomic study of the gut microbiota of wild Atlantic salmon (*Salmo salar*) (Rasmussen *et al.*, 2023), a high relative abundance of *Mycoplasma* was consistently observed across individuals from different regions and exposed to various environmental conditions, suggesting strong host-driven selection. In contrast, the lack of dominant *Ureaplasma* in the gut microbiota of captive whale sharks in the present study implies that *Ureaplasma* is more dependent on environmental factors than on host-specific factors. Although further studies need to focus on the isolation and culture of *Ureaplasma* from whale sharks, these bacteria residing in the whale shark gut may function to break down urea into ammonia during extended fasting periods in order to recycle nitrogen, or alternatively, may possess the ability to degrade chitin derived from planktonic organisms.

In the present study, fecal samples collected from two newly captured whale sharks up to 74 and 77 days after their transfer to the tank were assumed to still reflect the microbiota of wild individuals. However, wild individuals placed in captivity inevitably experience environmental changes, which may prevent their microbiota from completely reflecting the wild environment. Therefore, future studies need to collect samples before these changes occur. For example, fecal samples may be taken from the cloaca using swabs immediately after capture.

Overall, the present study provides valuable insights into differences in the fecal microbiota between newly captured and long-term captive whale sharks. The results obtained suggest that starvation markedly affects the fecal microbiota of whale sharks migrating along the Japanese coast, highlighting the impact of ecological and behavioral factors on‍ ‍the microbial community composition. Additionally, *Ureaplasma* identified in the gastrointestinal tract of whale sharks may represent a novel species. Further research is needed to clarify its taxonomic position and physiological roles, particularly in nitrogen recycling and chitin degradation in the host’s gut. Advances in this research may offer a novel approach to conservation by enabling ecological and behavioral inferences about wild whale sharks based on their fecal microbiota. Moreover, further ana­lyses of the dynamically changing microbiota of whale sharks during their long-term captivity, the diet they consume, and their health status will help promote our understanding and improve animal husbandry practices. These efforts are expected to enhance the health management and welfare of captive whale sharks, contributing to their long-term care and conservation.

## Citation

Ito, T., Segawa, T., Takasaki, K., Matsudaira, T., Kiyatake, I., Irino, H., and Nakajima, Y. (2025) Comparison of the Fecal Microbiota from Long-term Captive and Newly Captured Whale Sharks (*Rhincodon typus*). *Microbes Environ ***40**: ME25023.

https://doi.org/10.1264/jsme2.ME25023

## Supplementary Material

Supplementary Material 1

Supplementary Material 2

## Figures and Tables

**Table 1.** Data on individual whale sharks (*Rhincodon typus*) sampled in this study.

Group	Individuals	Sex	Capture date	Capture period prior to sampling (day)	Fecal sampling period	Number of samples	Feed type^1^	Average (range) feed quantity (kg day^–1^)	Whale length at sampling (m)
Long-term captive	Shark A	Female	Aug. 25, 2014	1,700	Apr. 21, 2019– Nov. 5, 2019	8	K, M, A	6.5 (0.0–7.1)	5.8
	Shark B	Male	Jun. 16, 2015	1,410	Apr. 26, 2019– Sep. 27, 2019	5	K, M, W	7.0 (0.0–7.5)	5.5
	Shark C	Male	Jun. 13, 2018	326	May 1, 2019– Nov. 11, 2019	7	K, M, S, W	4.0 (0.0–5.5)	3.5
	Shark D	Male	Jun. 7, 2018	328	May 5, 2019– Nov. 2, 2019	5	K, M, S, W	5.8 (0.0–7.0)	3.8
Newly captured	Shark E	Female	Jun. 14, 2019	2	Jun. 16, 2019– Nov. 12, 2019	9	K, M, S, W	3.4 (0.0–5.5)	4.1
	Shark F	Male	Sep. 3, 2019	10	Sep. 13, 2019– Nov. 16, 2019	3	K, M	4.6 (0.0–5.0)	4.2

^1^ K: krill; M: mysid shrimp; S: Sakura shrimp; W: whitebait; A: artificial feed for elasmobranchs.
