# Supplementary material for: Comparison of the Fecal Microbiota from Long-term Captive and Newly Captured Whale Sharks (Rhincodon typus)
Source: Microbes Environ. 2025 Sep 30;40(3):ME25023. doi: 10.1264/jsme2.ME25023 (PMC12501868; doi:10.1264/jsme2.ME25023)
Supplement: Supplementary file 1 — Supplementary Material 1 [file 40_25023_s1.pdf]

Supporting Information. Comparison of the Fecal Microbiota from Long-term Captive and Newly Captured Whale Sharks (*Rhincodon typus*). Takaomi Ito, Takao Segawa, Kazuto Takasaki, Takahiro Matsudaira, Itsuki Kiyatake, Hiroyuki Irino, Yu Nakajima, Microbes and Environments.

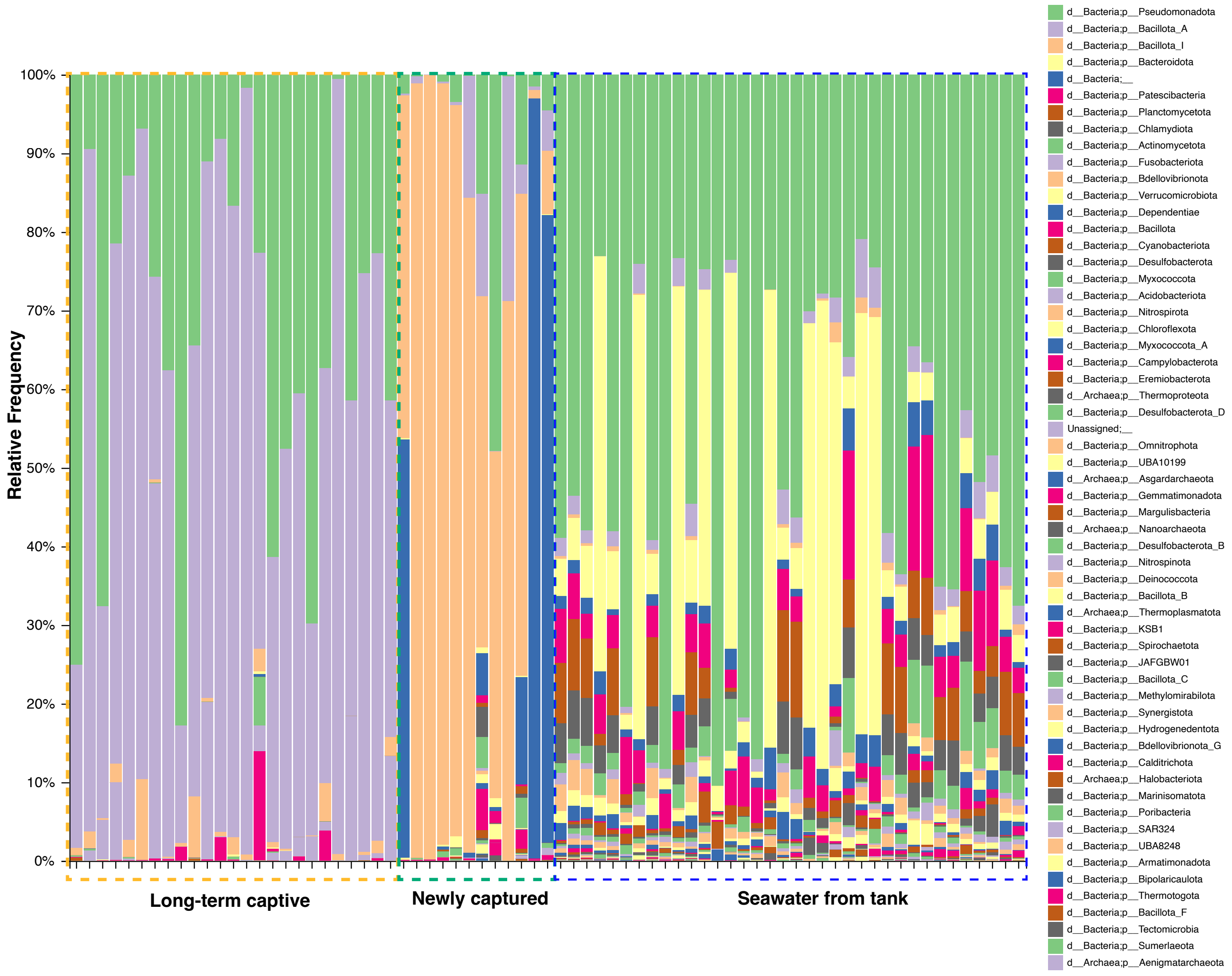

Supplementary Figure S1. Relative abundance of microbiota in fecal and seawater samples at the phylum level using all ASVs. Each bar represents each sample.

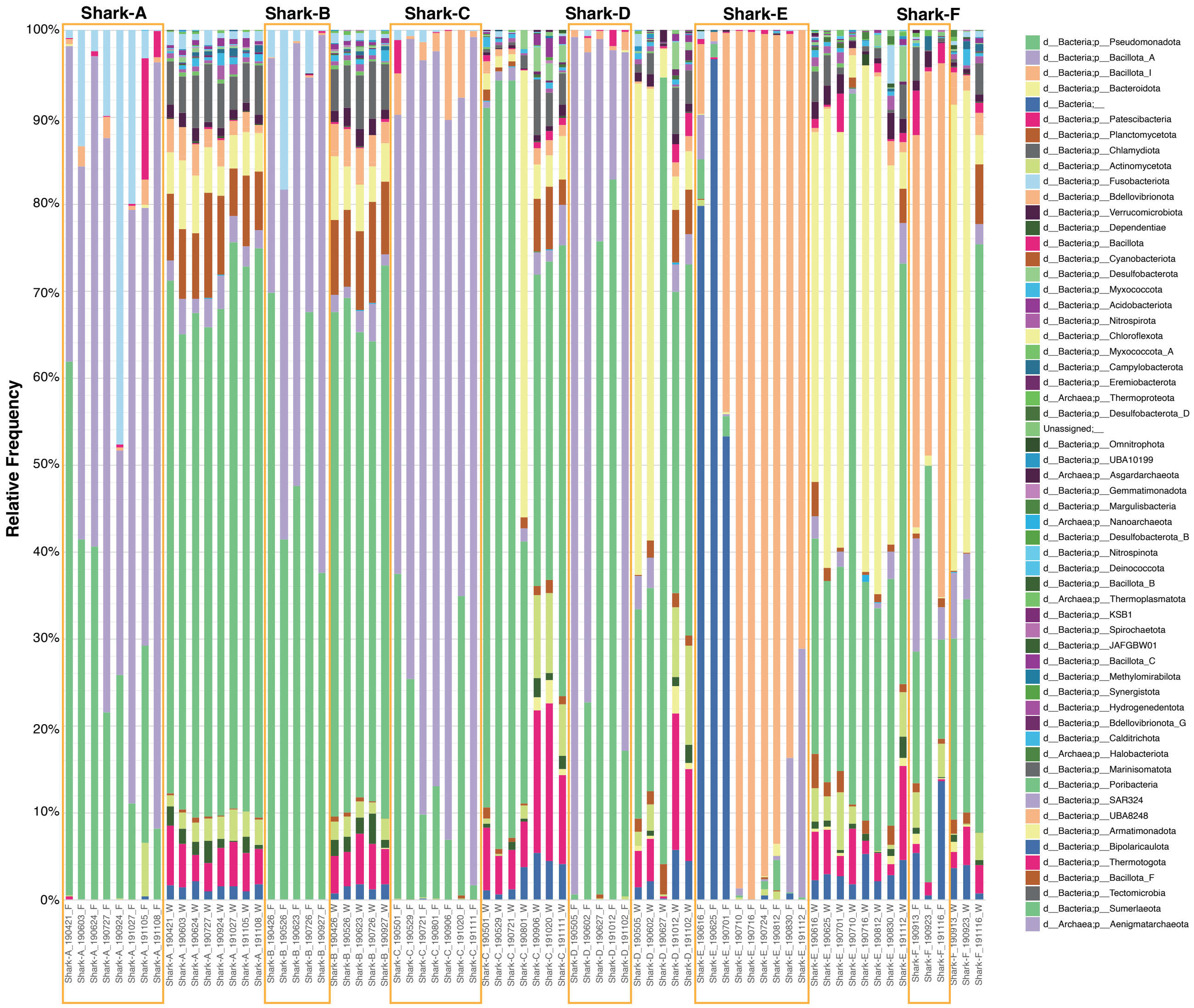

Supplementary Figure S2. Temporal change of microbiota on each individual at the phylum level. Each bar represents each sample, and the sample name indicates the individual's label (Shark-A to F), date of collection, and whether it was fecal (F) or seawater (W). The orange boxes means the range of fecal samples.
